# Pharmaco-proteogenomic profiling of pediatric diffuse midline glioma to inform future treatment strategies

**DOI:** 10.1038/s41388-021-02102-y

**Published:** 2021-11-10

**Authors:** Izac J. Findlay, Geoffry N. De Iuliis, Ryan J. Duchatel, Evangeline R. Jackson, Nicholas A. Vitanza, Jason E. Cain, Sebastian M. Waszak, Matthew D. Dun

**Affiliations:** 1 University of Newcastle, Cancer Signalling Research Group, School of Biomedical Sciences and Pharmacy, College of Health, Medicine & Wellbeing, Callaghan, NSW Australia; 2 Hunter Medical Research Institute, Cancer Research Program, New Lambton Heights, NSW Australia; 3 University of Newcastle, Reproductive Science Group, School of Environmental and Life Sciences, College of Engineering, Science and Environment, Callaghan, NSW Australia; 4 Ben Towne Center for Childhood Cancer Research, Seattle Children’s Research Institute, Seattle, WA USA; 5 Division of Pediatric Hematology/Oncology, Department of Pediatrics, Seattle Children’s Hospital, Seattle, WA USA; 6 Hudson Institute of Medical Research, Clayton, VIC Australia; 7 Department of Paediatrics, Monash University, Clayton, VIC Australia; 8 Centre for Molecular Medicine Norway (NCMM), Nordic EMBL Partnership, University of Oslo and Oslo University Hospital, Oslo, Norway; 9 Department of Pediatric Research, Division of Pediatric and Adolescent Medicine, Rikshospitalet, Oslo University Hospital, Oslo, Norway

**Keywords:** CNS cancer, Paediatric cancer

## Abstract

Diffuse midline glioma (DMG) is a deadly pediatric and adolescent central nervous system (CNS) tumor localized along the midline structures of the brain atop the spinal cord. With a median overall survival (OS) of just 9–11-months, DMG is characterized by global hypomethylation of histone H3 at lysine 27 (H3K27me3), driven by recurring somatic mutations in H3 genes including, *HIST1H3B/C* (H3.1K27M) or *H3F3A* (H3.3K27M), or through overexpression of *EZHIP* in patients harboring wildtype H3. The recent World Health Organization’s 5th Classification of CNS Tumors now designates DMG as, ‘H3 K27-altered’, suggesting that global H3K27me3 hypomethylation is a ubiquitous feature of DMG and drives devastating transcriptional programs for which there are no treatments. H3-alterations co-segregate with various other somatic driver mutations, highlighting the high-level of intertumoral heterogeneity of DMG. Furthermore, DMG is also characterized by very high-level intratumoral diversity with tumors harboring multiple subclones within each primary tumor. Each subclone contains their own combinations of driver and passenger lesions that continually evolve, making precision-based medicine challenging to successful execute. Whilst the intertumoral heterogeneity of DMG has been extensively investigated, this is yet to translate to an increase in patient survival. Conversely, our understanding of the non-genomic factors that drive the rapid growth and fatal nature of DMG, including endogenous and exogenous microenvironmental influences, neurological cues, and the posttranscriptional and posttranslational architecture of DMG remains enigmatic or at best, immature. However, these factors are likely to play a significant role in the complex biological sequelae that drives the disease. Here we summarize the heterogeneity of DMG and emphasize how analysis of the posttranslational architecture may improve treatment paradigms. We describe factors that contribute to treatment response and disease progression, as well as highlight the potential for pharmaco-proteogenomics (i.e., the integration of genomics, proteomics and pharmacology) in the management of this uniformly fatal cancer.

## Introduction

### Diffuse midline glioma

Diffuse midline glioma (DMG) is a devastating high-grade glioma (HGG) responsible for 50% of all childhood HGGs [1]. DMG is most frequently diagnosed in the brainstem (especially the pons, where it has historically been called diffuse intrinsic pontine glioma - DIPG), and less frequently in the midbrain, thalamus, and spine with patients presenting neurological symptoms such as cranial nerve deficits (facial asymmetry and diplopia), cerebellar signs (ataxia and dysarthria) and long tract signs (hyperreflexia and decreased strength) [2]. DMG patients face a very poor median overall survival (OS) of just 9–11-months, with <10% of patients with pontine tumors surviving two years post-diagnosis [3]. Due to the location within the critical structures of the brain or spine, and the diffuse and infiltrative growth characteristics of the tumor, significant surgical resection is extremely challenging to execute and most often impossible. This leaves radiotherapy as the only standard treatment; however, benefits are temporary for those who respond [4].

Investigations into the multitude of complex biological sequelae that underpin tumor formation and disease progression are key priorities in DMG. Landmark molecular profiling studies have identified numerous key genetic and epigenetic alterations, many co-segregating with age of onset, anatomical location, clinical outcome, histopathological and radiological features [1, 5, 6]. Investigations into the molecular pathogenesis of DMG has led to the classification of several molecular subtypes [1, 7]. The World Health Organization’s (WHO) 5^th^ Classification of Central Nervous System (CNS) Tumors, designates DMG as “diffuse midline glioma, H3 K27-altered” representing the majority of DIPGs as well as tumors found along the midline (e.g., brainstem, midbrain, thalamus, and spine). This classification encompasses molecular subtypes categorized according to alterations to lysine 27 in histone H3 (H3 K27-altered) [8], as well as patients harboring wildtype H3 and concomitant overexpression of the EZH inhibitory protein (*EZHIP*) [9].

### DMG histone H3 modifications

**Fig. 1:**
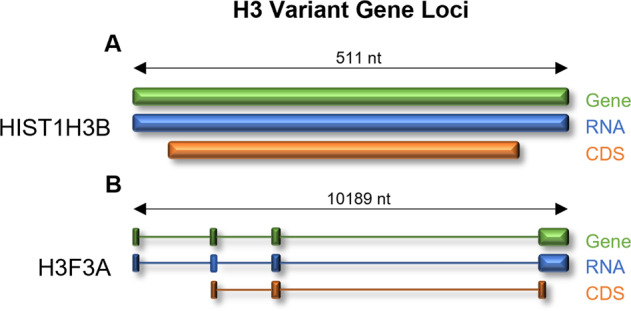
Gene-RNA-protein alignments of the mutant histone H3 genes that give rise to diffuse midline glioma. **A** The *HIST1H3B* (H3.1) gene is a short (511 nt) intronless gene, translated into a 136 amino acid, 15,404 (Da) protein. **B** Comparatively, the *H3F3A* gene is a long (10,189 nt), intron-containing gene, translated into a 136 amino acid, 15,328 (Da) protein.

The devastating transcriptional programs influenced by H3-alterations in DMG are fundamentally controlled by posttranslational modification (PTMs) of the 59 amino acid long N-terminal tail of H3, in both histone 3 isoform 1 (H3.1) and histone 3 isoform 3 (H3.3). These PTMs include acetylation, methylation, phosphorylation and ubiquitination and dictate protein structure, stability and accessibility, hence promotes or represses the activation of the transcriptional machinery complexes [10]. H3.1 variants are encoded by a cluster of intronless genes (HIST1 cluster), expressed in a replication dependent manner during the S-phase of the cell cycle [11]. Comparatively, H3.3 variants are independently encoded by two, intron-possessing genes (*H3F3A* and *H3F3B)* and are expressed throughout the cell cycle, however, are enriched at various stages of differentiation [12]. Of the genes encoding H3 histone variants, *HIST1H3B* (H3.1) and *H3F3A* (H3.3) harbor recurring mutations in DMG leading to the establishment of the molecular subtypes [9]. Between these two histone H3 genes, there are significant structural differences in both the presence of exon and introns, and nucleotide length (Fig. 1A, B), with only 3.4% genomic homogeny. However, the resultant histone proteins show 98.5% homogeny (pairwise sequence alignment using the EMBOSS needle alignment tool [13]). Key lysine residues such as K27 and K36 are conserved and play pivotal roles in the epigenetic regulation of transcription.

## Diffuse midline glioma, H3 K27-altered

### H3.1K27M- and H3.3K27M- DMG

Histone 3 lysine 27 to methionine (H3K27M) mutations occur in both H3.1 and H3.3 histone variants and are mutually exclusive. H3.1K27M is identified in 12–19% of DMG cases, with a median OS rate of 15 months, while H3.3K27M is identified in 65% of cases, with a median OS of 9 months [14]. H3K27M mutations are only translated into 3–17% of the total H3 protein pool [15], however, cause global loss of histone 3 lysine 27 trimethylation (H3K27me3) in the remaining wildtype H3 protein, leading to gene silencing normally regulated by the polycomb repressive complex 2 (PRC2) complex [16]. In healthy cells, the PRC2 complex auto-methylates its core subunits including the Suppressor of zeste 12 protein homolog (SUZ12) and two mutually exclusive and interchangeable catalytic subunits, Enhancer of zeste homolog 1/2 (EZH1/EZH2). Auto-methylation of PRC2 subunits increase histone methyltransferase activity by promoting accessibility to H3 tails where the catalytic pocket of PRC2 can methylate H3K27. Importantly, auto-methylation of EZH2 at K514me3 is reduced in cells transduced with H3K27M with a concomitant reduction in H3K27me2/me3 [17]. DMG harbor even more of a profound loss of EZH2-K514me3 and H3K27me2/me3, indicative of the reduced intrinsic activity of PRC2 [17]. Although the mechanistic basis for the loss of EZH2 methylation and hence PRC methyltransferase activity is not unequivocally resolved, structural studies indicate that the H3K27M mutant peptide shows affinity for EZH2 which may sequester or alter the conformation of the complex [18, 19]. Alternatively, H3K27M may impair the spread of repressive marks, that are preferentially retained at unmethylated CpG islands, affecting lowly-expressed genes influencing neurogenesis [15]. Nevertheless, global hypomethylation of H3K27 inhibits gene silencing and cell differentiation while promoting proliferation and is accompanied by synchronous co-enrichment in elevated H3K36me2, methylated by the Nuclear receptor binding SET domain protein 1/2 (NSD1/2) [20].

As the dominant molecular feature, treatment strategies targeting H3K27M are a priority, however, it has remained undruggable to-date. Given the associated loss of trimethylation and hence increased H3K27 acetylation (H3K27ac), it is of some surprise that most research has focused on the use of histone deacetylase (HDAC) inhibitors (HDACis), such as panobinostat, rather than inhibitors of histone acetyltransferases (HATs). Nevertheless, HDACis show low nanomolar cytotoxicity against DMG cell cultures and are effective in DMG patient-derived xenograft (PDX) mouse models [21, 22]. The HDACis, valproic acid, panobinostat, quisinostat and romidepsin, induce a dose-dependent global increase in H3K27ac and H3K27me3 [21–23], suggestive of a partial rescue of global H3K27 hypomethylation. This is consistent with findings that polyacetylation at residues both proximal and distal to K27M can greatly diminish PRC2 inhibition. Unfortunately, HDACi-induced partial rescue of global hypomethylation is transient [24], encouraging exploration into chemicals that synergize with HDACi. One promising candidate that is in preclinical development is the proteasome inhibitor marizomib which invokes acute toxicity through uncoupling of respiration and inhibition of glycolysis leading to metabolic catastrophe in DMG cells [25].

### Wildtype H3 K27 EZHIP-DMG

DMG cases harboring wildtype H3 is seen in approximately 10–15% of cases, with a median OS of 15 months, similar to H3.1K27M DMG [5]. Characterized by the overexpression of the *CXorf67* gene which encodes EZHIP [26], EZHIP-overexpressing DMG are a recent addition to the new WHO DMG subtype classification system [27]. EZHIP overexpression occurs in most wildtype H3 DMG cases [26], showing the ubiquitous nature of global H3K27me3 hypomethylation in DMG. A C-terminal peptide in EZHIP mimics the amino acid sequence of H3K27M, sequestering or altering the conformation of PRC2, reducing its histone methyltransferase activity [28]. Inhibition of PRC2 activity leads to an aberrant enrichment of H3K27ac marks and transcriptional programs remarkably analogous to H3K27M [19]. While direct inhibitors of EZHIP are yet to be developed, HDACis, such as panobinostat and quisinostat, show preclinical activity in wildtype H3 DMG, specifically those found in the pontine regions of the brain and that harbor a high mutational burden [22].

## DMG somatic and clonal heterogeneity

**Fig. 2:**
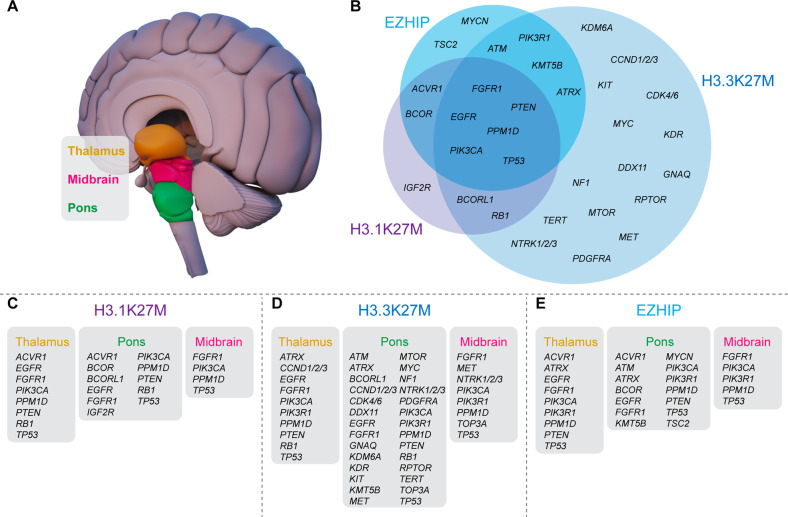
H3-altered diffuse midline glioma recurrent somatic mutations associated with each midline localization. **A** Most frequently, diffuse midline glioma (DMG) is localized in the pons (green), midbrain (pink) and thalamus (orange). **B** Venn diagram of recurrent somatic mutations seen in each H3-altered subtype, H3.1K27M (purple), H3.3K27M (light blue) and EZHIP (sky blue). Identity of recurrent somatic mutations in **C** H3.1K27M, **D** H3.3K27M and **E** EZHIP DMG H3-altered. Genomic information obtained by examining the comprehensive data published by whole-exome and whole-genome studies, references are including in Table 1.

We are gaining a greater understanding of the somatic heterogeneity of DMG [1], however, what is less understood is the impact of distinct genomic subclonal populations [6], potentially underpinning the lack of effective treatments. While recurring genetic H3-alterations are hallmark features of DMG, multiple co-segregating mutations are patient-specific, conferring their own varying level of poor prognoses and midline localization (Table 1, Fig. 2). The genomic landscapes of DMG have been comprehensively characterized [1], and in most cases, highlight the alterations that harbor potential for therapeutic targeting [29]. Even though we are yet to translate these discoveries into improved outcomes, these sophisticated real-time studies are providing us with increased knowledge of the co-occurring somatic events that underpin the genomic heterogeneity of DMG, critical information for the design of effective combination treatment strategies. To aid in the development of such combination strategies, in the following sections we summarize recurring somatic mutations linked with each DMG a subtype, list the recurrent midline localizations of these driver gene alterations (Fig. 2), and highlight potential therapies and research priorities that we hope will help to increase the durability and effectiveness of strategies targeting these mutations.

**Table 1:** Recurring genomic and proteomic alterations in diffuse midline glioma.

Genetic Alteration	Mutated Sites	Molecular Subtype	Prevalence	Location	Treatment	Reference	Section
*ACVR1*	R206H, G328V, G328W	H3.1K27M, EZHIP	32%	Pons, thalamus	LDN212854	[27, 46, 92, 93]	Activin receptor type-1 (*ACVR1*)
*ATM*	G2342V, L2877F	H3.3K27M, EZHIP	6%	Pons	AZD1390	[27, 43, 94]	ND
*ATRX*	H2254R, R2197L, L1357fs	H3.3K27M, EZHIP	10%	Pons, thalamus	Pyridostatin	[27, 49, 93]	ND
*BCOR*	C1363fs, A535V, G101fs	H3.1K27M, EZHIP	8%	Pons		[27, 93–95]	ND
*BCORL1*	S425I, R21H	H3.1K27M, H3.3K27M	7%	Pons		[1, 95]	ND
*CCND1/2/3*	Amplification	H3.3K27M	15%	Pons, thalamus	Palbociclib, ribociclib, abemaciclib	[43]	G1/S-specific cyclin-D2 (CCND2) / Cyclin-dependent kinases 4 and 6 (CDK4, CDK6)
*CDK4/6*	Amplification, L185V	H3.3K27M	15%	Pons	Palbociclib, ribociclib, abemaciclib	[1]	G1/S-specific cyclin-D2 (CCND2) / Cyclin-dependent kinases 4 and 6 (CDK4, CDK6)
*DDX11*	R186W, R167T	H3.3K27M	6%	Pons	Irinotecan	[49]	ND
*EGFR*	R108K, Amplification, CNG	H3.1K27M, H3.3K27M, EZHIP	4%	Pons, thalamus	Gefitinib, erlotinib	[1, 5, 14, 49, 96]	ND
*FGFR1*	K697E, N98S, N546K, K656E	H3.3K27M, H3.1K27M, EZHIP	12.5%	Pons, thalamus, midbrain	AZ4547, dovatinib, PD173074, ponatinib	[27, 93, 97]	ND
*GNAQ*	T96S	H3.3K27M	6%	Pons	Tris DBA palladium	[49]	ND
*IGF2R*	K162R, D1830E	H3.1K27M	8%	Pons	GSK1838705A	[68]	ND
*KDM6A*	Deletion, CNL	H3.3K27M	6%	Pons		[49, 94]	ND
*KDR*	S1154P, Amplification, CNG	H3.3K27M	4.8%	Pons	Mebendazole	[1, 49, 98]	ND
*KIT*	T96P, Amplification, CNG	H3.3K27M	4.8%	Pons	Mebendazole	[1, 49, 98]	ND
*KMT5B*	R187*, M646fs	H3.3K27M, EZHIP	1%	Pons	Olaparib, talazoparib	[6]	ND
*MET*	P664P, Amplification	H3.3K27M	10%	Pons, midbrain	Cabozantinib	[5, 49]	ND
*MTOR*	A1971V	H3.3K27M	1%	Pons	Everolimus, fimepinostat, AZD2014	[1, 55, 99]	Phosphatidylinositol-4,5-bisphosphate 3-kinase signaling cascade (PIK3CA/PIK3R1/PTEN/MTOR)
*MYC*	R33C, Amplification	H3.3K27M	12%	Pons	Omomyc	[1, 57]	MYC proto-oncogene protein (MYC)/ MYCN proto-oncogene protein (MYCN)
*MYCN*	Amplification, CNG	EZHIP	8%	Pons	Bromodomain inhibitors	[14, 68]	MYC proto-oncogene protein (MYC)/ MYCN proto-oncogene protein (MYCN)
*NF1*	G295R, R1204W, Deletion	H3.3K27M	10%	Pons	Binimetinib, trametinib	[1, 14, 95, 99]	ND
*NTRK1/2/3*	TPM3_NTRK1 VCL_NTRK2 ETV6_NTRK3	H3.3K27M	3.7%	Pons, midbrain	Larotrectinib	[5, 98]	ND
*PDGFRA*	Y288C, C235Y, Amplification	H3.3K27M	30%	Pons	Crenolanib, dasatinib	[1, 42, 93]	Platelet derived growth factor receptor alpha (*PDGFRA*)
*PIK3CA*	E545K, I391M, H1047R	H3.3K27M, H3.1K27M, EZHIP	12%	Pons, thalamus, midbrain	Paxalisib, fimepinostat	[43, 55, 93]	Phosphatidylinositol-4,5-bisphosphate 3-kinase signaling cascade (PIK3CA/PIK3R1/PTEN/MTOR)
*PIK3R1*	K567E, G376R	H3.3K27M, EZHIP	18%	Pons, thalamus, midbrain	Paxalisib, everolimus, fimepinostat	[27, 43, 49, 55]	Phosphatidylinositol-4,5-bisphosphate 3-kinase signaling cascade (PIK3CA/PIK3R1/PTEN/MTOR)
*PPM1D*	W427*, E525X, Q404X, E405X, 428 fs	H3.1K27M, H3.3K27M, EZHIP	25%	Pons, thalamus, midbrain	CCT007093, GSK2830371, olaparib	[27, 53, 70]	Protein phosphatase, Mg2+/Mn2+dependent 1D (PPM1D)
*PTEN*	A126S, R130X, Deletion	H3.1K27M, H3.3K27M, EZHIP	19%	Pons, thalamus	Fimepinostat	[7, 27, 43, 49, 53, 55]	Phosphatidylinositol-4,5-bisphosphate 3-kinase signaling cascade (PIK3CA/PIK3R1/PTEN/MTOR)
*RB1*	Amplification, Deletion	H3.1K27M, H3.3K27M	16%	Pons, thalamus		[14, 97]	ND
*RPTOR*	D857N	H3.3K27M	1%	Pons		[1, 43]	ND
*TERT*	C228T, C250T	H3.3K27M	2%	Pons	Imetelstat	[5]	ND
*TOP3A*	C633Y	H3.3K27M	3–4%	Pons, midbrain	PIP-199	[1]	ND
*TP53*	G245S, R175H, R248Q, R248W, R273C, R273H, S241F, V157F	H3.1K27M, H3.3K27M, EZHIP	60–80%	Pons, thalamus, midbrain	APR-246, GSK-J4	[1, 27, 30]	Cellular tumor antigen p53 (TP53)
*TSC2*	D1587V, Q1035*	EZHIP	2%	Pons	Rapamycin	[1]	ND

*ND* not determined.

### Cellular tumor antigen p53 (*TP53*)

Cellular tumor antigen p53 (*TP53)* is the second most recurring lesion in H3.3K27M DMG (60-80%) [30] (Table 1, Fig. 2B). *TP53* mutations are also seen in H3.1K27M and EZHIP DMG, however, considerably less frequently (13.3% and 11.1% respectively) [1, 27]. *TP53* is a tumor suppressor, encoding the p53 protein which transmits a variety of stress-inducing signals to different antiproliferative cellular responses including apoptosis, senescence, and cell-cycle arrest [31]. Mutations in the *TP53* gene are known to lead to tumor immortality through epigenetic dysregulation and elimination of H3K27me3-driven oncogene repression [32]. These mutations are also the main driver of increased radiotherapy-resistance in DMG, both in patients and corresponding cellular models [33]. The introduction of loss of function (LoF) mutations in the *TP53* gene and knock-in of H3.3K27M mutations are enough to induce neural stem cell self-renewal in mice. This occurs via transcriptional and epigenetic control of the proliferative genes necessary to drive DMG formation in vivo and are further exacerbated by knock-in of activating mutations in platelet derived growth factor receptor alpha (*PDGFRA*, discussed in “Platelet derived growth factor receptor alpha (*PDGFRA*)”) [34].

The recurrent nature of *TP53* mutations highlight the importance of therapies that act as surrogate regulators of apoptosis and cell cycle arrest; however, this has been notoriously difficult to achieve. Examining the functional outcomes of *TP53* mutations which result in either a partial or full distortion to the DNA-binding domain, has led to the development of the prodrug, APR-246. Upon activation, APR-246 binds to and stabilizes mutant p53, reactivating the protein and driving tumor suppression in preclinical models [35]. In addition to p53 reactivation, APR-246 also elevates reactive oxygen species (ROS) production through dysregulation of redox systems. This propels apoptotic elements and drives oxidative DNA damage, increasing genotoxic stress [36]. The efficacy of APR-246 is further enhanced by radiotherapy (~50%), and when combined with the Jumonji demethylase inhibitor GSK-J4 [30], increased survival of preclinical models [37, 38].

As *TP53* LoF mutations are challenging to treat, targeting cell proliferation/survival signaling pathways activated uniquely in response to *TP53* mutations, may be an alternative therapeutic approach. Tyrosine phosphoproteomic profiling of *TP53*-mutant mouse tumor models, revealed unique up-regulation of the proto-oncogene, receptor tyrosine kinase (RTK) Mesenchymal–epithelial transition factor (MET) [39]. Treatment of cultured p53-null cells exhibiting MET amplification with a selective MET tyrosine kinase inhibitor (TKI) (PHA-665752) abrogated aberrant tyrosine phosphorylation and blocked cell proliferation. MET inhibition has also shown preclinical efficacy in *TP53* mutant glioblastoma (GBM) cell lines and patient-derived GBM cells, particularly when combined with inhibitors of epidermal growth factor receptor (EGFR) [40]. These observations highlight a possible treatment option for *TP53* mutant DMG.

### Platelet derived growth factor receptor alpha (*PDGFRA*)

Activation of *PDGFRA* accelerates DMG formation in mice, with recurring mutations seen in 14.4% of DMG patients [41] and gene amplification in 30% of DMG, primarily H3.3K27M tumors [42] (Table 1, Fig. 2B). Platelet derived growth factor receptors (PDGFRs) influence cell migration, proliferation and survival with ligand induced receptor dimerization driving auto-phosphorylation and activation [43]. Transduction of downstream signals is regulated by a multitude of pathways, but predominately PI3K/Akt/mTOR [41, 43] (discussed in “Phosphatidylinositol-4,5-bisphosphate 3-kinase signaling cascade (PIK3CA/PIK3R1/PTEN/MTOR)”). Co-segregating with H3.3K27M, amplified *PDGFRA* strongly promotes glioma formation in vivo, resulting in a clinically aggressive form of DMG [41] (Fig. 2B). Tyrosine kinase inhibitors (TKIs) approved by the US Food and Drug Administration (FDA), such as dasatinib and crenolanib, target *PDGFRA*, however are cytostatic, not cytotoxic, and hence do not extend the survival of DMG patients [44] (Table 2). Deep proteomic, phosphoproteomic and transcriptomic profiling of *PDGFRA*-mutant HGG mouse models identified PI3K/Akt signaling as responsible for driving MYC (discussed in “MYC proto-oncogene protein (MYC)/ MYCN proto-oncogene protein (MYCN)”) and JUN (Transcription factor AP-1) activity [45], to create a positive-feedback loop increasing expression of multiple other RTKs leading to oncogene addiction. Importantly, the activity of oncogenic signaling pathways activated downstream of *PDGFRA* mutations were only visible via assessment of the phosphoproteome, highlighting the importance of the evaluation of the posttranslational architecture of DMG in the design effective treatment strategies.

**Table 2:** Clinical trials involving the use of targeted therapy drugs in DMG and other pediatric gliomas.

Treatment	Clinical Trial Identifier	Phase	Description	Posted results
Abemaciclib	NCT02644460	Phase I [Recruiting]	Clinical trial evaluating abemaciclib in patients with newly diagnosed or relapsed/refractory DMG aged 2–25-years.	
	NCT04238819	Phase I [Recruiting]	Study to determine the safety and efficacy of abemaciclib in combination with temozolomide and irinotecan in patients with relapsed/refractory solid tumors aged up to 18-years.	
AZD1390	NCT03215381	Phase I [Completed]	Study to analyze the PK of AZD1390 in healthy adult males aged between 20–65-years.	No results posted
	NCT03423628	Phase I [Recruiting]	Study to test safety, tolerability, and PK of AZD1390 and radiotherapy for the treatment of glioblastoma in patients aged18–130-years.	
AZD2014	NCT02619864	Phase I [Completed]	Clinical trial to determine MTD of AZD2014 in combination with temozolomide in glioblastoma patients 18- years and older.	No results posted
Binimetinib	NCT02285439	Phase I/II [Active, not recruiting]	Clinical trial to determine MTD of binimetinib (MEK162) in patients with low grade glioma aged 1–18-years.	
BMS-986158	NCT03936465	Phase I [Recruiting]	Study investigating the bromodomain inhibitor, BMS-986158, for brain tumors in patients aged 1-21-years.	
Cabozantinib	NCT02885324	Phase II [Recruiting]	Clinical trial to test cabozantinib for HGG patients aged 2–21-years.	
Crenolanib	NCT01393912	Phase I [Completed]	Phase I clinical evaluated crenolanib in patients with newly diagnosed DMG or in recurrent, progressive, or refractory HGG aged 18 months-21-years.	No results posted
Dasatinib	NCT01644773	Phase I [Completed]	Clinical trial to determine MTD of crizotinib and dasatinib for patients with DMG and other HGG aged 2–21-years.	No results posted
	NCT00996723	Phase I [Completed]	Clinical trial to evaluate the combination of vandetanib and dasatinib during and after radiotherapy in patients with DMG 18 months–21-years.	No results posted
Erlotinib	NCT01182350	Phase II [Terminated]	Clinical trial tested combinations of FDA-approved agents (including erlotinib) in patients with DMG aged 3–18-years based on specific biologic targets.	64.4% of patients had a 9-month overall survival rate following treatment
Erlotinib	NCT02233049	Phase II [Unknown]	Clinical trial comparing response of DMG patient to erlotinib, everolimus and/or dasatinib depending on biological abnormalities, aged 6 months–25-years.	
Everolimus	NCT03387020	Phase I [Completed]	This study examined the side effects and best dose of ribociclib and everolimus as well as how well they work in treating patients, aged 1-21-years, with recurrent/refractory DMG.	MTD for ribociclib and everolimus was determined to be 120 and 1.2 mg/m^2^/day respectively.
	NCT03355794	Phase I [Active, not recruiting]	Clinical trial examining the safety of ribociclib and everolimus, when administered to DMG patients aged 1-30-years following radiation therapy.	
	NCT03632317	Phase II [Withdrawn]	Clinical trial evaluated the activity of panobinostat in combination with everolimus for patients aged 2 to 30 years with newly diagnosed HGG or DMG after radiotherapy.	Low accrual
Fimepinostat	NCT03893487	Early Phase I [Recruiting]	Clinical trial studying the efficacy of fimepinostat in treating patients aged 3 to 39 years with newly diagnosed DMG	
Gefitinib	NCT00042991	Phase I/II [Completed]	Clinical trial studied the efficacy of gefitinib, in combination with radiation therapy, in treating patients aged 3-21 years with brainstem gliomas and glioblastoma.	This trial found median progression -free survival to be 7.43 months on average while overall survival was 12.12 months
	NCT00052208	Phase I/II [Completed]	Study investigated side effects and best dose of gefitinib when administered in conjunction with radiotherapy as well as its effectiveness in treating patients of all ages with glioblastoma.	No results posted
Imetelstat	NCT01836549	Phase II [Terminated]	Phase II clinical trial studied the efficacy of imetelstat in treating patients aged 12 months to 21 years with recurrent or refractory brain tumors.	Terminated due to several intracranial hemorrhages and recommendation by the PBTC DSMB.
Larotrectinib	NCT04655404	Early Phase I [Recruiting]	Clinical trial evaluating the disease status in patients aged up to 21 years with HGG with TRK fusion following larotrectinib treatment.	
Mebendazole	NCT01837862	Phase I/II [Recruiting]	Study determining the safety and efficacy of mebendazole in combination with chemotherapy drugs for the treatment of DMG in patients between 1 and 21 years of age.	
	NCT02644291	Phase I [Recruiting]	Clinical trial investigating the safety and side effects of mebendazole in patients aged 1-21 years for recurrent brain cancers that are no longer responsive to standard therapies.	
Olaparib	NCT03233204	Phase II [Recruiting]	Study observing the effectiveness of olaparib in treating patients (12 months to 21 years) with relapsed/refractory solid tumors, which possess defects in DNA damage repair genes.	
	NCT03155620	Phase II [Recruiting]	Phase II trial studying the efficacy of genetic testing-directed treatment (including the drug olaparib) in patients, between aged 12 months to 21 years, with advanced solid tumors.	
ONC201	NCT03416530	Phase I [Recruiting]	Multicenter, seven arm, dose escalation, clinical trial studying ONC201 in patients aged 2 to 18 years with DMG and recurrent/refractory H3 K27M gliomas.	
Palbociclib	NCT03709680	Phase I [Recruiting]	Study evaluating palbociclib in combination with chemotherapy in patents aged 2-20 years with medulloblastoma or DMG.	
Ponatinib	NCT02478164	Phase II [Completed]	Clinical trial studied ponatinib as a potential treatment for recurrent glioblastoma unresponsive to bevacizumab in patients aged 18 years and older.	This clinical trial had no patients with a 3-month progression free survival and an average overall survival of 98 days.
Rapamycin	NCT02420613	Phase I [Active, not recruiting]	Phase I trial studying the side effects and best dose of rapamycin (temsirolimus) when given together with vorinostat and with or without radiation therapy in patients aged 7 months to 21 years with DMG	
Ribociclib	NCT03355794	Phase I [Active, not recruiting]	Clinical trial examining the safety of ribociclib and everolimus, when administered to patients aged 12 months to 30 years with DMG following radiation therapy.	
	NCT02607124	Phase I/II [Terminated]	Study investigated the effects of ribociclib when given after radiation therapy in DMG patients aged 12 months to 30 years.	Terminated due to a competing trial opened for patient population with combination of ribociclib and everolimus.
	NCT03387020	Phase I [Completed]	Clinical trial examined the side effects and best dose of ribociclib and everolimus and their efficacy in treating patients aged 1-21 years with treatment resistant or relapsed malignant brain tumors, including DMG.	No results posted
Talazoparib	NCT04740190	Phase II [Recruiting]	Study testing the effectiveness of talazoparib in recurrent glioblastoma in patients aged 18 years and older.	
Trametinib	NCT03919071	Phase II [Recruiting]	Clinical trial examining how well dabrafenib and trametinib works after radiation therapy in HGG patients aged 3- 25 years.	

### Activin receptor type-1 (*ACVR1*)

Activin receptor type I (*ACVR1*) is mutated in approximately 32% of all DMG [32], 87% of H3.1K27M [1] and 72% (13/18 cases) of EZHIP [27] (Table 1, Fig. 2A–C). *ACVR1* encodes the serine/threonine protein kinase, activin receptor-like kinase-2 (ALK2), belonging to bone morphogenetic protein (BMP) signaling pathway, transforming growth factor-beta (TGF-β) superfamily. Activation of ALK2 regulates morphogenesis, differentiation, proliferation, and apoptosis during embryonic development [43]. ALK2 phosphorylation activates BMP signaling and leads to the phosphorylation and activation of the SMAD (mothers against decapentaplegic family) transcription factors [43]. *ACVR1* mutations lead to constitutive activation of BMP signaling, thus, activation of SMAD, driving expression of DNA-binding protein inhibitors, ID1 and ID2. This expression promotes tumor initiation and accelerates gliomagenesis whilst repressing differentiation [43, 46]. Somatic mutations in *ACVR1* are unique to DMG, with analogous germline mutations seen in fibrodysplasia ossificans progressiva (FOP). Currently, there are no curative treatments for *ACVR1* mutations in FOP, thus treatments have instead focused on the inhibition of BMP [43]. Preclinical compounds such as LDN-212184, have been developed to inhibit ALK2 and hence phosphorylation of SMAD, with pharmacokinetic (PK) studies showing sufficient brain penetration [46]. Although LDN-212184 extends survival of orthotopic DMG PDX mouse models, ALK2 inhibitors, with improved specificity and potency, are necessary if these treatments are to translate to better outcomes for DMG patients.

Given that the intracellular activity of SMADs is reliant on their phosphorylation, numerous protein phosphatases are known to downregulate their activity and may serve as targets for therapies to enhance ALK2 inhibition [47]. The serine/threonine protein phosphatase, PP2A is a SMAD-associated phosphatase frequently showing reduced activity in cancer, with activity increased using fingolimod (FTY720), an FDA approved drug used in the treatment of multiple sclerosis, and hence has excellent brain penetration [48]. This encourages preclinical assessment of FTY720 in combination with therapies targeting ALK2 for *ACVR1* mutant DMG.

### Phosphatidylinositol-4,5-bisphosphate 3-kinase signaling cascade (*PIK3CA/PIK3R1/PTEN/MTOR*)

Mutations in the components of the Phosphatidylinositol-4,5-bisphosphate 3-kinase (PI3K) signaling axis are recognized drivers of gliomagenesis in DMG [43]. Mutations in the PI3K catalytic subunit alpha (*PIK3CA*) are seen in 12% of DMG (Fig. 2), while PI3K regulatory subunit 1 (*PIK3R1*) mutations are present in 18%, most commonly in H3.3K27M and EZHIP subtypes [43, 49] (Table 1, Figs. 2B, C). *PIK3CA* mutations lead to constitutive lipid kinase activity thereby driving cellular transformation [50], while *PIK3R1* mutations activate wildtype *PIK3CA* or PI3K signaling [51]. Phosphatase and tensin homolog (*PTEN*) is the well-established negative regulator of this signaling cascade, and an important tyrosine kinase tumor suppressor [52]. *PTEN* is mutated in 4% of H3.1K27M [53], 6% of H3.3K27M [49], and 6% of EZHIP DMG [27] (Table 1, Fig. 2). Loss of PTEN can occur through a chromosomal deletion of 10q and is determined to be an early event in DMG development [52].

Paxalisib is an FDA approved, PI3K/Akt inhibitor developed to penetrate the brain and decrease activity of signaling cascades [54]. Recently, paxalisib was shown to reduce growth and proliferation in PI3K-mutant and wildtype DMG cell lines [54] and has recently entered combination clinical trials for DMG (NCT05009992). Fimepinostat (CUDC-907), is a dual PI3K/HDAC inhibitor (Table 2), that inhibits radiation-induced DNA repair pathways including homologous recombination and nonhomologous end-joining, leading to G1 cell-cycle arrest and apoptosis [55] and is also in DMG clinical trials (NCT03893487). Despite PI3K alterations being some of the common events in DMG and other cancers, pharmacological inhibition of PI3K has resulted in variable clinical responses. This raises the possibility of an inherent mechanism of resistance. Indeed, mouse tumor models show insulin feedback is induced by PI3K inhibitors, reactivating PI3K signaling, thus compromising their efficacy [56]. Insulin-feedback is effectively controlled using anti-glycemic approaches, which greatly enhances the therapeutic effectiveness of PI3K inhibitors, an approach that warrants rigorous testing in DMG.

### MYC proto-oncogene protein (*MYC*) / MYCN proto-oncogene protein (*MYCN*)

The MYC proto-oncogene transcription factor family, including MYCL proto-oncogene (MYCL), Cellular myelocytomatosis oncogene (c-MYC) and MYCN proto-oncogene (MYCN), are important mediators of many growth-promoting signal transduction pathways. *MYC* alterations are common in human cancers including DMG, reported in 20% of the H3.3K27M subtypes [57] (Table 1, Fig. 2B). Interestingly, the *MYCN* gene has been used to define a subset of DMG characterized by CpG hypermethylation, high-grade histology, and chromothripsis on chromosome 2p in tumors, leading to recurrent amplification of *MYCN* [7]. This subtype is seen in 8% of DMG, and predominately associated with EZHIP [7]. High MYC activity is correlated with poor outcomes, but it is very difficult to target owing to its ‘undruggable’ protein structure [57]. To overcome this, Omomyc, a peptide-based dominant negative inhibitor, was developed to outcompete MYC/MAX dimers for binding to E-box DNA sequences. MYC dimerizes with MAX to activate transcription and promote cell proliferation. Omomyc binds to MAX, blocking MYC binding, repressing MYC-mediated gene expression profiles in H3.3K27M DMG preclinical models [57, 58].

Hyperacetylation of H3K27 in DMG is driven by the activity of acetyl-binding, bromodomain and extraterminal (BET) proteins, particularly bromodomain-containing protein 4 (BRD4) which are implicated in tumor progression and aggressiveness [59]. MYC is highly occupied by H3.3K27M and H3K27ac super-enhancers and hence highly expressed in DMG. This is slightly at odds with what has been previously shown, where *MYC*/*MYCN* amplification was only observed in EZHIP DMG [7]. Panobinostat decreased oncogenic *MYC* target gene expression causing cell death in DMG preclinical models [21].

Targeting BRD4-driven MYC activity, using the BBB-penetrant bromodomain inhibitor, JQ1, is effective as a monotherapy in EZHIP DMG models when used at high-doses, however, H3K27M DMG are less sensitive [23]. Combinations of JQ1 and MRK003, a gamma-secretase inhibitor that reduces *NOTCH1* expression, reduced H3K27M DMG growth and survival [60], highlighting this as a potential strategy for DMG.

### G1/S-specific cyclin-D2 (*CCND2*) / cyclin-dependent kinases 4 and 6 (*CDK4*, *CDK6*)

G1/S-specific cyclin-D2 (*CCND2*) functions as a regulator of Cyclin-dependent kinase 4 and 6 (*CDK4*/*CDK6*) which contributes to the temporal coordination of the cell cycle [61], and typically altered in H3.3K27M DMG (Table 1, Fig. 2B). Activation of the cyclin/CDK complex leads to the hyperphosphorylation of the Retinoblastoma (RB) tumor suppressor protein and dissociation from the transcription factor E2F1. The recruitment of HATs and subsequent transcription of E2F1 target genes advance mitotic progression through the G1/S phase [61]. Modulation of cyclins such as CCND2 (G1/S-specific cyclin-D2) occurs in nearly all tumors with the amplification of *CCND2* being a frequent genomic event in DMG and predominates in tumors of the pontine region [1]. CDK4/6 mutations are also common in DMG, highlighting the potential of targeting the cyclin signaling axis. In melanoma, activating mutations in *CDK4* abolish interactions with the tumor suppressor Cyclin dependent kinase inhibitor 2 A’s (CDKN2A) / p16^INK4A^, rendering the protein constitutively active, leading to uncontrolled cell cycling [62].

Given the importance of cyclin proteins in mitosis, several CDK4/6 inhibitors have been developed and are under clinical evaluation in DMG [63] (Table 2). Palbociclib has completed dose escalation studies, while two other trials are testing ribociclib. Abemaciclib is suggested to be the most effective CCND2-CDK4/6 inhibitor due to higher CDK4/6 affinity/potency compared to palbociclib and ribociclib [63]. While these CDK4/6 inhibitors have shown some efficacy in tumors with deregulated cell-cycle control, resistance to these therapies is common. Phosphoproteomic profiling of cancer models resistant to CDK4/6 inhibitors revealed enhanced Mitogen-activated protein kinase (MAPK) signaling, therapeutically exposed using the FDA approved MEK inhibitor, trametinib [64] (Table 2). Targeting MEK is a treatment paradigm that has recently come into clinical thinking particularly for HGGs and DIPGs harboring germline or somatic mutations in Neurofibromatosis type 1 (*NF1*) [65, 66]. Furthermore, the combination of MEK1/2 and CDK4/6 inhibition showed therapeutic synergy across a broad panel of high-risk neuroblastoma preclinical models [67], a treatment paradigm that warrants exploration in DMG.

### Protein phosphatase, Mg^2+^/Mn^2+^ dependent 1D (*PPM1D*)

Activating mutations in the Protein phosphatase, Mg^2+^/Mn^2+^ dependent 1D (*PPM1D*) are seen in 15% of H3.3K27M [68], 3.8% of H3.1K27M [68], and 11.1% of EZHIP [27] DMG (Table 1, Fig. 2). *PPM1D* mutations inactivate ATM- (Ataxia telangiectasia mutated protein) and ATR- (Ataxia telangiectasia and Rad3-related protein) mediated DNA damage response (DDR) and dephosphorylate H2A.X and p53 in response to radiation [69]. Knockdown of *PPM1D* is almost curative in DMG PDX models [70]. Hence an allosteric, non-competitive inhibitor of *PPM1D*, GSK2830371, has been developed, which drives p53-dependent inhibition of DMG tumor growth [70]. Additionally, GSK2830371 sensitized *PPM1D*-mutant DMG to PARP inhibition using olaparib (Table 2). Although olaparib showed no brain penetration against intact BBB, tumor penetration was detected in orthotopic GBM xenografts, penetrating both tumor core and tumor margins of GBM patients with recurrent tumors [71]. Whether GBM’s tumor vasculature characteristics (leaky vasculature) translate to olaparib uptake in DMG is yet to be reported.

### Intertumoral and intratumoral heterogeneity

The somatic alterations summarized above offer some biological insight into the roles these recurring mutations play in DMG and present us with a suite of therapeutic vulnerabilities. Despite the number of currently used targeted therapies developed to combat the various co-occurring mutations in DMG, most are still undergoing early-stage clinical trials to establish toxicity profiles. More recent studies are now using them in combination, however, the maximum tolerated doses for these combinations are yet to be determined, and efficacy signals are unknown (Table 2).

**Fig. 3:**
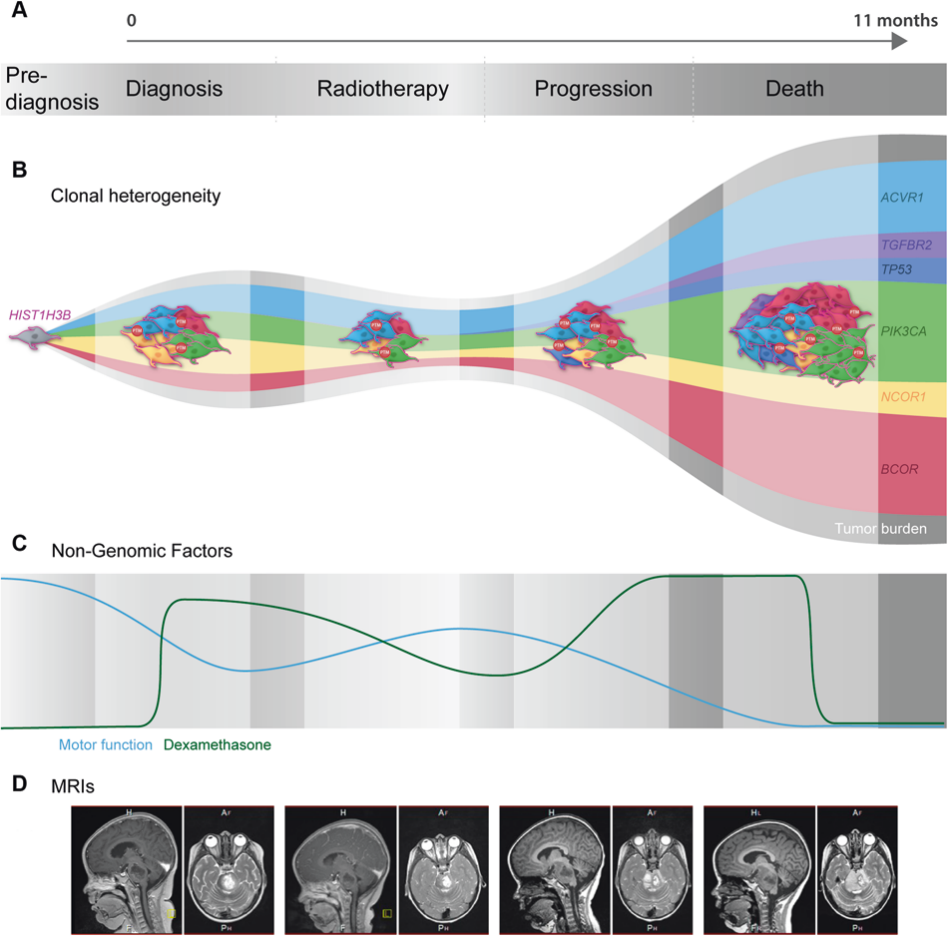
Clonal evolution, tumor burden and non-genomic contributions to diffuse midline gliomas development and progression. **A** Diffuse midline glioma (DMG) tumor burden continually increases following diagnosis. Similarly, the clonal heterogeneity of DMG evolves throughout a patient’s disease, potentially influenced by endogenous and exogeneous factors, including microsatellite instability, treatment, and steroids. **B** Representation of tumor evolution beginning with a single tumor cell harboring a *HIST1H3B* mutation, cell outlined in pink. As the tumor grows and diversifies, it gains new driver and passenger mutations. Driver mutations in this example include *ACVR1* in light blue, *TGFBR2* in purple, *TP53* in dark blue, *PIK3CA* in green, *NCOR1* in yellow and *BCOR* in red. **C** In addition to the increased clonal heterogeneity and tumor burden, non-genomic factors fluctuate throughout disease progression and likely contribute to growth and survival. The patient’s degree of motor function, represented by a blue line, is inversely proportional to tumor burden, whereas corticosteroids anti-inflammatory medications (dexamethasone), represent by a green line, is relatively proportional to tumor burden beginning with a sharp increase at diagnosis, sustained then decreased during radiotherapy, and adjusted to meet the patient’s symptoms [100]. **D** Representative magnetic resonance imaging (MRI) of tumor development and progression throughout a DMG patient’s journey.

The complexity of these clonal cancer genomes emphasizes why a one-drug-fits-all model has unequivocally failed patients. Furthermore, tumors typically have multiple mutations of unknown significance, making it challenging to ascertain which of these genetic lesions are primary oncogenic drivers. Investigation into the effectiveness of drug combinations as means to combat the somatic heterogeneity of DMG has been performed in multiple DMG models using high-throughput drug screening, and comprehensive molecular analysis to correlate drug sensitivities with genomic influences [21, 25]. While the intertumoral heterogeneity of DMG is well established and exploited to develop treatments (Table 1, Table 2), the intratumoral diversity of this cancer represents a relatively novel field in which to better understand DMG. Analysis of nonsilent mutations in DMG revealed high intratumor (clonal) heterogeneity as a proportion of nonslient mutations [53], the extent of which is greater than or equal to all other cancers [72]. In both DMG and pediatric GBMs, multiple somatic subclones co-exist, both spatially and temporally [6, 44]. These co-existing tumor subclones are suspected to play important tumorigenic roles in response to CNS active therapies and may enhance proliferative, tissue invasion and oncogenic signaling, to promote tumor cell dissemination. Indeed, DMG subclones co-cultured or transplanted together, enhanced the invasion into surrounding tissues of less-mobile colonies [44]. These tumor subclones, populated by varying co-segregating driver and passenger mutations, continually evolve alongside tumor burden during a patient’s clinical journey (Fig. 3). Targeting of one of these subclonal driver mutations may show success in combating that oncogenic colony, however, genomic diversity between subclones encourages their spread and indeed diversification, ultimately leading to resistance. Hence targeted therapies provide limited therapeutic benefits [73]. The growth and diversification of these subclones influence, and are influenced by, non-genomic factors including the tumor microenvironment (TME), concurrent corticosteroid therapy, growth factors, stress hormones and electrical signals [74] that increase invasiveness and provide a survival advantage, as well as potentiate intratumoral heterogeneity [75] (discussed further in “Phosphoproteomic and tumor microenvironmental influences”).

## Moving towards systems biological approach

### Germline analysis

To progress treatment and improve outcomes we need to take a systems-wide view of DMG and employ multi-omics approaches to develop treatments, starting with the analysis of both the somatic and germline mutations. Characterization of a patient’s germline and somatic variants, coupled with understanding of the systems-wide response to therapies, will potentiate the personalization of drug-dose and -timing, as well as the selection of the most appropriate therapy for each patient. Germline DNA analysis is increasingly becoming as important as somatic evaluation. Not only does it aid in distinguishing key somatic events, but also identifies any potential inherited influences that may play an important role in drug PK and pharmacodynamic (PD) disposition. For example, patients carrying Uridine 5’-diphospho-glucuronosyltransferase (UGT) polymorphisms have impaired ability to inactivate 7-ethyl-10-hydroxycamptothecin (SN-38), the active metabolite of irinotecan, and hence face significant toxicities. If polymorphisms are known, patients receive reduced dose [76] which may affect response. This may be an important consideration in the case of DMG response to radiotherapy, as common *TP53* germline mutations may influence response [33].

It is important to consider that gene expression profiles rarely correlate with the abundance of the corresponding protein, influenced by a range of post -transcriptional / -translational effects. These effects include amino acid composition which impacts the rate of translation elongation [77], miRNA expression, and the battery of posttranslational modification (>200) that characterize the mammalian proteome [78]. This may provide us with some insight into why genomically-targeted, precision therapies are yet to provide a therapeutic benefit for DMG patients. A quantitative trait loci (QTL) is a region of DNA associated with a particular phenotypic trait that varies dependent on polygenic effects such as expression of multiple genes, and the environment in which they are expressed; while protein quantitative trait loci (pQTLs) provide a functional readout to identify how genetic variants regulate protein expression; necessary to reveal the proteome’s role in disease causation [79]. QTL mapping using somatic mutations, germline variants and sensitivity to 265 drugs, in 993 cancer cell lines, identified 78 drugs with at least one significant genetic association (drug response QTL). Remarkably, nine of these genetic associations involved germline variants, comparable with effects related to somatic variants, highlighting that germline variants contribute to protein abundance, a key factor in modeling drug sensitivity and response [80]. Given germline mutations in cancer-predisposing genes are seen in 9% of non-DMG pediatric cancers [81], it is likely that these mutations have a yet-to-be-determined role in the gliomagenesis and treatment resistant characteristics of DMG. While genomic sequencing provides a static snapshot of the cellular environment, these studies are limited in their ability to interpret the contribution of genetic features to the biology of patient’s tumor, leaving clinicians with therapies that offer little hope of efficacy. The cancer proteome, however, can narrow the gap between genotype and phenotype, providing a more appropriate platform for studying the kinetics of drug response, as it accounts for the plasticity and dynamic nature of cancer cells and, when assessed using tumors treated in vivo, can help to reveal the important contribution of the TME [82].

### Phosphoproteomic and tumor microenvironmental influences

**Fig. 4:**
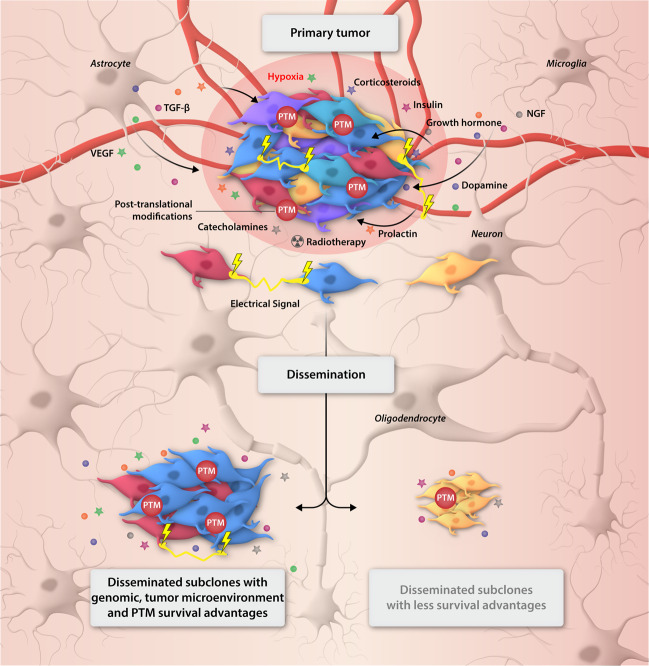
Non-genomic contributions to diffuse midline glioma growth and progression. Diffuse midline gliomas (DMG) are vastly complex tumors localized in the midline structures of the brain. At diagnosis (primary tumor) DMG harbor numerous driver mutations, (highlighted by tumor cells of varying color), that contribute to drive the evolution of the cancer. Surrounding cells and structures of the midline of the brain such as blood vessels, neurons, astrocytes, microglia, and oligodendrocytes contribute to the gliomagenesis of DMG, whether through direct physical connections or by more indirect mechanisms, such as electrical signals (yellow lightening blots) between glioma and normal cells or the contribution of growth hormones, NGF, VEGF, TGF-β, and prolactin, or even, endogenous factors, such as hypoxia, dopamine, insulin, catecholamines. The extracellular cues drive posttranslational modifications (PTMs) that influence the activity of oncoproteins that contribute to the aggressive nature of the disease. It is likely that exogenous factors, such as radiotherapy (radioactive symbol) and corticosteroids (dexamethasone), also contribute to the disease, the impact on tumor growth and treatment resistance yet to be fully understood. The diffuse and infiltrative growth of this cancer also leads to dissemination throughout the brain. Disseminated subclones, however, can differ in genomic and proteomic characteristics to that of the primary tumor, influenced by clonal selection supported by non-genomic factors from each different region of the brain, and lead to distinct survival and proliferative advantages, highlighting the challenge we face in developing treatment strategies that will lead to long-term survival for patients diagnosed with DMG.

Global assessment of the PTMs that influence the activity of oncoproteins that ultimately drive gliomagenesis in DMG is now necessary. Coupled with molecular and pharmacological information, assessment of the proteome and posttranslational architecture of DMG reveal the proteomic heterogeneity of DMG in response to treatment. While some DMG harbor constitutively activated RAS/MAPK pathways [57], not all cells within the tumor will show this signal-type. Proteomic heterogeneity is not always a simple consequence of the heterogeneity of the genome and is always affected by endogenous factors, including metabolic and neurological cues, and exogenous stressors such as steroids, experimental treatments and radiotherapy [57]. The TME itself is a complex network of cells, organelles and structures such as blood vessels, neurons, astrocytes, microglia, and oligodendrocytes and filled with endogenous factors, including catecholamines such as dopamine, insulin, growth hormones, factors likely to influence tumor development and progression [57] (Fig. 4).

Interestingly, but perhaps not surprisingly, another microenvironmental influence on DMG tumor progression is neuronal activity. Electrochemical communications occur between gliomas and neurons through synapses and drive proliferation, differentiation, and survival (Fig. 4). Neuronal excitability and thus the release of growth factors promote glioma propagation [74]. A common characteristic of the TME is the infiltration of immune cells which influence the behavior of tumor cells. In adult GBMs, infiltrating immune cells stimulate tumor proliferation and invasion. Tumor-associated macrophages originate from microglia and/or bone marrow-derived monocytes to drive cellular migration and metastasis and release pro-inflammatory cytokines to promote a glioma stem-like state [83]. However, primary DMG display little immune cell infiltration when compared to GBM and other low-grade gliomas (LGG). These findings reveal minimal immune activation in the DMG TME, the impact of which is currently unclear [44]. These microenvironmental influences are vital to tumor development and, when acting in conjunction proteomic heterogeneity, aids in the natural selection of aggressive tumor subclones [72] (Fig. 4). For instance, this can occur by changing the milieu of certain conditions and/or growth factors following therapy. The hypoxic microenvironment that remains following radiotherapy typically favors tumor cells harboring activated HIF1α, aiding survival and poor outcomes [84].

While these microenvironmental factors likely influence tumor progression and the effectiveness of treatments, systems-wide analysis can be used to predict drug efficacy. Indeed, proteomics has been acknowledged as one of the most effective predictors of drug cytotoxicity compared to gene copy number, mutations, and methylation analysis, which provide poor predictors of the sensitivity of human cancer cell lines to an extensive list of oncology and non-oncology drugs [85].

### Pharmaco-proteogenomics

There are clear benefits to integrating complementary pharmacogenomic and pharmacoproteomic research when investigating new treatment paradigms for patients. Techniques such as single nucleotide polymorphism (SNP) arrays, whole genome sequencing (WGS) and RNA-sequencing (RNA-seq) should be implemented in conjunction with high-throughput quantitative proteomics [86, 87], validated via high-throughput multiplex immunohistochemical imaging and TME analysis using limited tissue from biopsy. This will provide us with a more effective target prediction platform, and lead to more durable responses [88].

Large-scale, comprehensive proteogenomic analysis in pediatric brain cancers has recently been performed using 218 tumors across 7 histological types with thousands of proteins and phosphoproteins, correlated with mutations and copy number variations (CNVs). Of these pediatric CNS tumors, 25 were HGGs, 10 of which were localized to the midline [89]. Proteomic and phosphoproteomic profiling analyzed by consensus clustering revealed eight distinct subtypes, used to predict survival outcomes, proliferation indices and pathway activation, that spanned histological boundaries. Of the 10 HGG localized to midline structures, 7 subtyped within the *HGG-rich* cluster, while 3 were grouped in LGG BRAF^Fusion^-rich, Ganglioglioma-rich and Ependymoma clusters. Kinase enrichment analysis using HGG phosphoproteomes, revealed an elevated abundance of phosphorylated substrates of CDK1 and CDK2. CDK2 shared kinase-substrate association with Minichromosome maintenance complex component 2 (MCM2) at Ser 139 and Nucleophosmin (NPM1) at Ser 70, with MCM2 and NPM1 vital for the regulation of cell proliferation, suggesting CDK2 plays a dominant role in promoting DMG cell proliferation, and hence a target of worthy pursuit. An independent signaling node in HGG was the Calcium/calmodulin dependent protein kinase II alpha (CAMK2A) also shown to be the most abundant protein in gangliogliomas; but in HGG a higher correlation between kinase activity and protein abundance was evident [89], highlighting the importance of simultaneous analysis of the transcriptome/proteome and the phosphoproteome. CAMK2A in association with Gap junction protein alpha 1 (GJA1) phosphorylated at Ser 325 and Ser 314, plays an important role in metastatic invasion, promoting gap junction assembly between glioma cells and surrounding astrocytes [90] as well as increasing synaptic transmission through the phosphorylation of Synapsin-1 (SYN1) at Ser 605 by CAMK2A [89]. Given the limited number of DMG samples profiled in this study, it is currently unknown whether these two mutually exclusive HGG proliferation (CKD2-MCM2-NPM1) and invasion (CAMK2A-GJA1-SYN1) oncogenic signaling pathways are associated with specific DMG H3-subtypes, highlighting the necessity for similar studies assessing the phospho-proteogenomic characteristics of DMG, studies that are ongoing in our laboratory. Nevertheless, the observation that the phosphorylation status of these pathways dictates their oncogenic function, which remains invisible to genomic approaches, highlights the importance of a systems-wide view to aid in the development of effective treatment strategies for DMG patients.

Importantly, not only can pharmaco-proteogenomics offer a functional molecular readout of the potential of therapeutic targets but can also be used to refine molecular subtypes based on drug response. Quantitative proteomics coupled with RNA sequencing led to the development of a distinct binary classification system for Isocitrate dehydrogenase 1/2 *(IDH)* wildtype GBM. These tumors were clustered based on elevated protein expression of either FKBP9 (Prolyl isomerase 9), or PHGDH (Phosphoglycerate dehydrogenase) and RFTN2 (Raftlin family member 2) [91]. Tumors with high expression of FKBP9 correlated with poorer prognosis *IDH* wildtype GBMs, while high expression of PHGDH and RFTN2 provided a more favorable prognosis. Integration of these proteomic subtypes with pharmacological profiles using matched patient-derived GBM cells showed mTORC1/2 inhibition (AZD2014) as an efficient strategy for patients harboring poor prognostic biomarkers. This highlights the power of pharmaco-proteogenomics to uncover treatment targets that guide therapeutic interventions that could possibly be used to provide more of a functional readout than simply subtyping DMG based on H3 K27-alterations [8].

Naturally, there are challenges associated with employing ‘multi-omics’ techniques in DMG. Genomics is challenged by poor drug responses, defining candidate genes, reproducibility, statistical analysis and the requirement to interrogate large datasets of polymorphisms in large numbers of patients [87], while proteomics is challenged by the need for large amounts of starting material, which is almost impossible to obtain at biopsy, complex protocols and study design, with clinical sites needing appropriate infrastructure, expertise and robust analytical tools to ensure successful execution [88].

It is now a key challenge to try and integrate CNS pharmacokinetics into chemotherapeutics and pharmaco-proteogenomics to design regimens that will benefit patients. Treatments based solely on genomic prediction are limited due to the posttranscriptional and posttranslational architecture of DMG, which are yet to be fully elucidated. Genomics has become an essential element of sophisticated clinical trials [29] with genome data from tumor biopsies and/or blood plasma being used to assign the tumor to a molecular subtype and to detect genetically-distinct tumor subclones. Despite this, genomic data alone does not always provide the required insights into a patient prognosis or treatment options that deliver improved patient outcomes. Coupling phosphoproteomic approaches with in-depth genomic analyses will help to identify recurrent genetic alterations and their associated protein-controlled functional outcomes, the therapeutic potential of which can be furthered by the integration of pharmacological studies to accelerate novel clinical trials with biomarkers of prognostic and predictive value. In summary, sophisticated imaging and nuclear medicine should guide resection of multiple biopsy samples from representative regions of the tumor to help overcome regional clonal heterogeneity which will aid in the prediction of beneficial therapies, particularly when genomics is coupled with proteomics which will additionally aid in the assessment of regional contributions of the TME and immune system.

## Conclusion

Despite extensive developments in novel targeted therapies and precision medicines, the prognosis and outcomes of patients diagnosed with DMG remain unacceptably poor. The recent 5th Edition of the WHO Classification of Tumors of the CNS, subtypes DMG based on H3 K27-alterations and facilitates the categorization of patients according to distinct clinicopathological and molecular features. It is important to note that the hallmark H3-alterations that give rise to DMG are somewhat unique to these tumors; therefore, novel modalities targeting these alterations herald our greatest chance to improve treatment. However, long-term successful outcomes will require treatments that take into appreciation the yet-to-be-characterized proteomic heterogeneity of DMG, including the assessment of the posttranslational architecture. Furthermore, future studies focused on regional contributions to tumor growth and survival are also needed as are studies to determine the mechanisms that influence immune system avoidance. Until genomics-based treatment target identification is integrated with pharmacogenomics and pharmacoproteomics research, the success of trials will remain low, with little hope of patients achieving long-term survival. Coupled evaluation of the DMG genome with the respective proteome, will enhance treatment selection/development, refine the evaluation of patient prognosis, and lead to the development, we hope, of approaches that improve outcomes for those diagnosed with the most aggressive, and poorly survived pediatric cancer.
